# Mobile-collector capture of particles in a chaotic flow

**DOI:** 10.1371/journal.pone.0329766

**Published:** 2025-08-07

**Authors:** Mengying Wang, Julio M. Ottino, Paul B. Umbanhowar, Richard M. Lueptow

**Affiliations:** 1 Department of Mechanical Engineering, Northwestern University, Evanston, Illinois, United States of America; 2 Department of Chemical and Biological Engineering, Northwestern University, Evanston, Illinois, United States of America; 3 Northwestern Institute on Complex Systems (NICO), Northwestern University, Evanston, Illinois, United States of America; Universiti Malaysia Pahang Al-Sultan Abdullah, MALAYSIA

## Abstract

Removing dispersed material, such as pollutants, from dynamic fluid environments like the ocean or the atmosphere is challenging when the flow is chaotic. Here the capture of passive tracer particles by a mobile collector (MC) is studied in a model two-dimensional chaotic flow with vortices. Four simple capture strategies for determining the MC direction are considered, all of which rely on periodic measurement of the local particle distribution. The ultimate success of a strategy depends on its associated motion and detection parameters as well as the underlying fluid flow. When the flow is fully chaotic or the relative velocity of the MC is large, the four strategies exhibit nearly equal effectiveness. However, when the flow is less chaotic and the relative MC velocity is small, the collector can become trapped in or outside of a vortex. Changing the particle detection parameters can prevent trapping, which improves capture. In the absence of trapping and for both high and low relative velocities of the MC, a scaling analysis explains the dependence of the capture rate on the relevant dimensionless variables based on timescales for the mobile collector and the underlying flow. For a wide range of parameters and all four capture strategies, the capture timescale depends linearly on a combination of the characteristic kinematic timescale related to the relative motion of the collector and the gradient timescale related to the underlying flow field, confirming that the capture process is properly characterized.

## Introduction

The flow capture problem focuses on removing dispersed material from fluid flows, with the most significant and pressing applications involving pollutant removal from geophysical flows of water and air [1–3]. Stationary plants are well suited for removing atmospheric pollution, where CO_2_ remediation is arguably the most urgent problem [4,5], and pilot plants for direct carbon capture have been put into service in Switzerland [6] and Iceland [7]. Since the atmospheric mixing time is short, on the order of one year [8], compared to the expected duration of CO_2_ removal, on the order of decades, stationary carbon capture plants offer an appropriate approach to capturing and, thereby, reducing atmospheric CO_2_.

In contrast to atmospheric remediation, removing pollutants from the surface of lakes and oceans is often best achieved using *mobile collectors*, such as ships upon which skimmers that suck or scoop up contaminants such as trash or oil from the water surface can be deployed. For context, every year millions of tons of plastic debris pour into the ocean and circulate with the ocean surface currents to form large accumulation zones, such as the Great Pacific Garbage Patch [9] which has been the target of recent cleanup efforts [10,11]. Another application of mobile flow capture is mitigating water-borne hazards, such as oil from the Deepwater Horizon spill, which caused significant damage to the environment in and around the Gulf of Mexico [12] when millions of barrels of crude oil leaked from a damaged oil well, of which only a small portion was ultimately captured.

To achieve efficient and rapid removal of pollutants using flow capture requires a better and more sophisticated understanding of how to best move a mobile collector (MC) through a flowing pollutant field. Motivation for investigating flow capture with an MC comes from oil spill mitigation, which typically uses response vessels to detect and capture spilled oil. For instance, Grubesic, *et al*. [13] proposed an application of advanced oil spill modeling techniques combined with a mathematical model to optimize offshore oil spill cleanup resulting in the removal of 95% of the spilled oil in various scenarios. In another study, García-Garrido, *et al*. [14] combined dynamical systems theory and remote sensing techniques to analyze the 2015 Oleg Naydenov fishing trawler oil spill, which successfully reproduced the true ocean movement on two particular days.

Barker, *et al*. [15] offers a comprehensive review of current progress in modeling oil spill response and remaining challenges. Related recent research has focused on oil spill modeling (see Keramea, *et al*. [16] for a review) and the optimization of resource utilization and specific decision-making tools and strategies under a variety of conditions including oil spill detection, cold water effects, etc. [17,18] without considering the details of clean-up. Other studies include some aspects of clean-up such as deploying mobile skimmers [19] or other response vessels [20] but without delving into the fluid transport aspects of the clean-up strategy. In one case, strategic operations for oil spill response vessels is considered [21], but again without considering dynamics effects of fluid transport of the oil. While these previous studies provide guidance for predicting and forecasting oil spills and subsequent strategies for their clean-up, this paper focuses on characterizing the influence of specific MC motion strategies and properties on capture of tracer particles in chaotic fluid flows typical of ocean surfaces.

A major challenge of solving the flow capture problem is the complex spatio-temporal variation of geophysical flows like those in the atmosphere and the ocean. However, general features of geophysical flows can be realized in simpler model flows that share similarities with ocean and atmospheric flows such as large-scale vorticity and chaotic flow. In order to avoid the complexity of real geophysical flows while focusing on fundamental aspects of the flow capture problem, a two-dimensional model chaotic flow that includes large-scale vortical structures and a degree of chaos that can be varied by changing the model parameters is considered. In particular, this work considers the chaotic double-gyre flow model [22], which exhibits similar dynamical properties to real geophysical flows [23,24] and has been previously used to characterize the capture capability of a stationary collector (SC) [25,26]. This research is also inspired by autonomous surface and underwater vehicles, which are used for collecting scientific data from the ocean and often require path planning in general flows with limited energy budgets [27–30]. Most closely related to the present work, Senatore, *et al*. [31] tracks a theoretical vehicle in the double-gyre flow considered here and demonstrates that the most fuel-efficient trajectories move along the Lagrangian coherent structures of the flow.

In this paper, the chaotic double-gyre flow model is used to study tracer particle capture with a single mobile collector. First, a capture algorithm is introduced which uses local information from the vicinity of the MC for four different strategies for choosing the motion direction. Then the effectiveness of the MC in removing particles from the flow for varying flow conditions and MC parameters is considered. Conditions under which the MC can become trapped in sub-regions of the flow are described and how this unwanted trapping can be avoided is discussed. Last, when the MC is untrapped, the scaled characteristic capture time is well-characterized by two distinct mechanisms depending on the relative velocity of the MC to the flow. We note that this study is restricted to passive rather than active dispersed materials, see [32,33]. For example, carbon dioxide molecules can be regarded as passive; however, some water-borne debris, may not be, with the formation of the Great Pacific Garbage patch being a rather distressing example of aggregation driven by the interactions of its constituents.

## Double-gyre flow model

**Fig 1:**
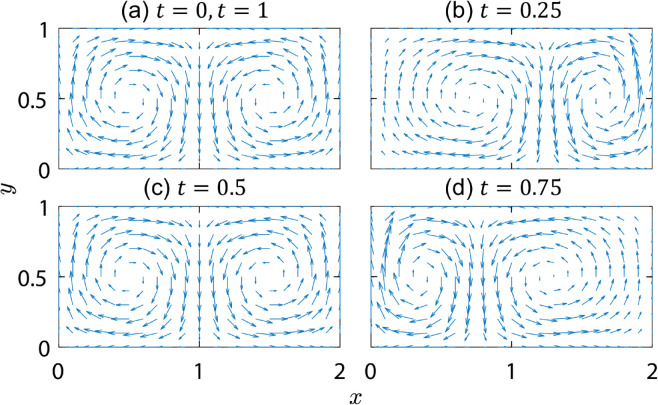
Time dependence of the velocity field of the double-gyre flow (Eq 1) during one period with ω=2π, A=π/2 and ϵ=0.25.

The velocity field for the double-gyre flow model [22], which is used here as a simplified model of geophysical flow, is given by

$$v_{x}(x,y,t)=-A\sin[\pi f(x,t)]\cos(\pi y),$$ (1a)

$$v_{y}(x,y,t)=A\cos[\pi f(x,t)]\sin(\pi y)\frac{\partial f}{\partial x}(x,t),$$ (1b)

$$f(x,t)=\epsilon \sin(\omega t)x^{2}+[1-2\epsilon \sin(\omega t)]x,$$ (1c)

where *A* is a velocity amplitude, and *ε* and ω/2π control the amplitude and frequency of the temporal flow variation, respectively. (Note that in order to simplify the notation and without any loss of generality, the velocity amplitude is written as *A* instead of πA as in previous studies [22–26].) The flow is limited to a bounded 2×1 rectangular region, and time is measured in units of the flow period, T=2π/ω. When A=π/2≈1.5, the domain-averaged flow velocity is approximately one, independent of the value of *ε*. The velocity field at different times during one flow period with ϵ=0.25 is illustrated in Fig 1. The flow consists of two counter-rotating vortices (gyres) that complete one side-to-side oscillation within one flow period, *T*. The value of *ε* specifies the maximum side-to-side displacement of the vertical line separating the vortices along which $v_{x}=0$. At *t* = 0, or any integer or half-integer of the period, the vortices are symmetric. At other times, the $v_{x}=0$ line is displaced from *x* = 1 as it oscillates in the *x*-direction with right and left extremes occurring at *t* = 0.25 (corresponding to *T*/4) and *t* = 0.75 (corresponding to 3*T*/4), respectively. The combination of *A*, *ε*, and *ω* determine the chaotic character of the flow as discussed later in the Scaling Section.

**Fig 2:**
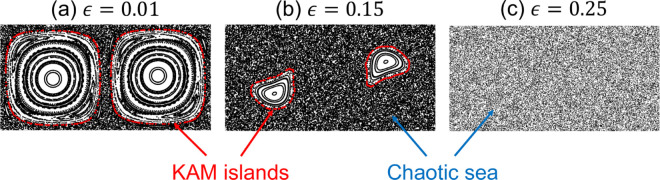
Poincaré map of double-gyre flow with ω=2π, A=π/2 and various *ε* in the 2×1 flow domain.

A standard technique for visualizing transport of tracer particles in the double-gyre model is the Poincaré map, shown in Fig 2 for A=π/2 and ϵ∈{0.01,0.15,0.25}. The map is generated from the positions of tracer particles (points) initially uniformly distributed across the domain and then plotted at equal interval integer flow periods [34]. Tracer particles initially located in non-chaotic flow regions form what are known as KAM (Kolmogorov-Arnold-Moser) islands [35–37], each of which contain at least one elliptic point [32]. Tracer particles initially located in chaotic regions explore the entire “chaotic sea." In Fig 2(a) for ϵ=0.01, there is a relatively small chaotic sea (regions with no evident structure), and two large non-chaotic KAM islands, the position and shape of which are time-dependent [25]. As *ε* is increased, the KAM islands decrease in size [Fig 2(b)], and eventually the flow domain becomes fully chaotic [Fig 2(c)]. In addition, there can be leaky barriers to mixing at the edges of the KAM islands called Cantori [38,39], which become important to the capture process for SCs when the overall capture times are long [25].

Smith, *et al*. [26] note that the Poincaré map reflects the capture fraction of individual SCs at particular locations in the flow. A collector in the chaotic sea will capture all tracer particles in the chaotic sea. In contrast, a collector within a KAM island captures only the subset of tracers within that island having trajectories that intersect the collector. However, an SC located in the chaotic sea for one portion of the flow period and in a KAM island for the other portion eventually captures all tracers in the chaotic sea as well as some tracers from the island. This time- and position-dependent character of the flow capture problem with a stationary collector is considered in substantial detail to determine optimal capture locations in our previous paper [25].

Motivated by this previous research on stationary collectors [25,26], mobile collectors are considered here using the same approach of advecting tracer particles in the double-gyre flow for a range of flow and MC parameters. Tracer particles are removed from the flow when their trajectories intersect the perimeter of the collector. Moving the collector through the flow, as is done in this paper, increases the likelihood that its path intersects both chaotic regions and non-chaotic islands, which should improve its capture capability.

## Capture simulations and strategies

The central challenge in implementing an effective MC strategy is prescribing the collector motion. Here, our approach is inspired by a bacterial foraging algorithm for dynamic environments [40]. In the foraging algorithm, two options are considered at each time step, either continue along in the same direction if the food along the current path is sufficient or switch to a random alternate direction if there is not enough local food. A similar algorithm to move a collector in the chaotic double-gyre flow with the goal of efficiently capturing tracer particles is implemented here.

In this study, a circular MC is characterized by two parameters: its diameter, *δ*, which equals 0.1 except as noted, and its velocity magnitude relative to the surrounding fluid, $v_{rel}$. The resulting lab frame (domain) velocity, ${\vec{v}}_{MC}$, is the sum of the MC relative velocity and the local fluid velocity, $\vec{v}(x,y)$:


$${\vec{v}}_{MC}=\vec{v}(x,y)+{\vec{v}}_{rel}.$$

**Fig 3:**
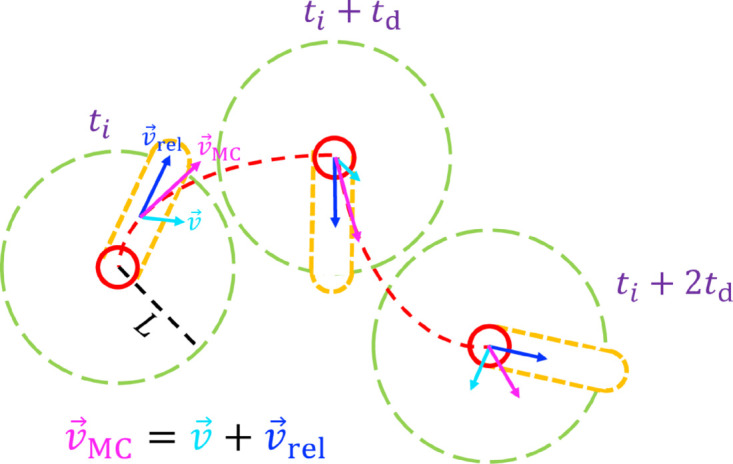
Schematic of algorithm for moving a collector in a chaotic flow (see text for full description).

The MC trajectory relative to the base flow is then determined by the direction of ${\vec{v}}_{rel}$. Here, four different strategies are explored to control the direction of ${\vec{v}}_{rel}$, all of which share the same two-step procedure shown schematically in Fig 3. The first step at time *t*_*i*_ examines a circular region of radius, *L*, around the MC with angular resolution of $3.6^{\circ }$ and then initially orients ${\vec{v}}_{rel}$ in the direction in which a straight, length *L* trajectory from the current MC position captures the most tracer particles in the absence of relative flow (i.e., the flow is considered to be frozen). In the second step, the MC moves for a finite duration, $t_{d}$, with relative speed $v_{rel}$ and an overall displacement relative to the fluid of $v_{rel}t_{d}$. The procedure is repeated at intervals of $t_{d}$ until the end of the simulation.

Three additional specifications are needed to fully define the MC motion algorithm: i) when no particles are observed in any direction, the MC continues to follow the direction associated with the current strategy; ii) if the edge of the *δ*-diameter collector contacts the boundary of the domain, the MC stops, repeats the observation process, and proceeds in a new direction for the remaining portion of the current $t_{d}$-duration motion interval, unless it again encounters the boundary whereupon it repeats the re-orientation procedure; iii) if multiple directions contain the same number of tracer particles during the observation step, the direction with the largest weighted sum $\sum \frac{1}{\Delta r_{i}}$ is chosen, where $\Delta r_{i}$ denotes the distance to particle *i* within the observation area associated with each direction.

**Fig 4:**
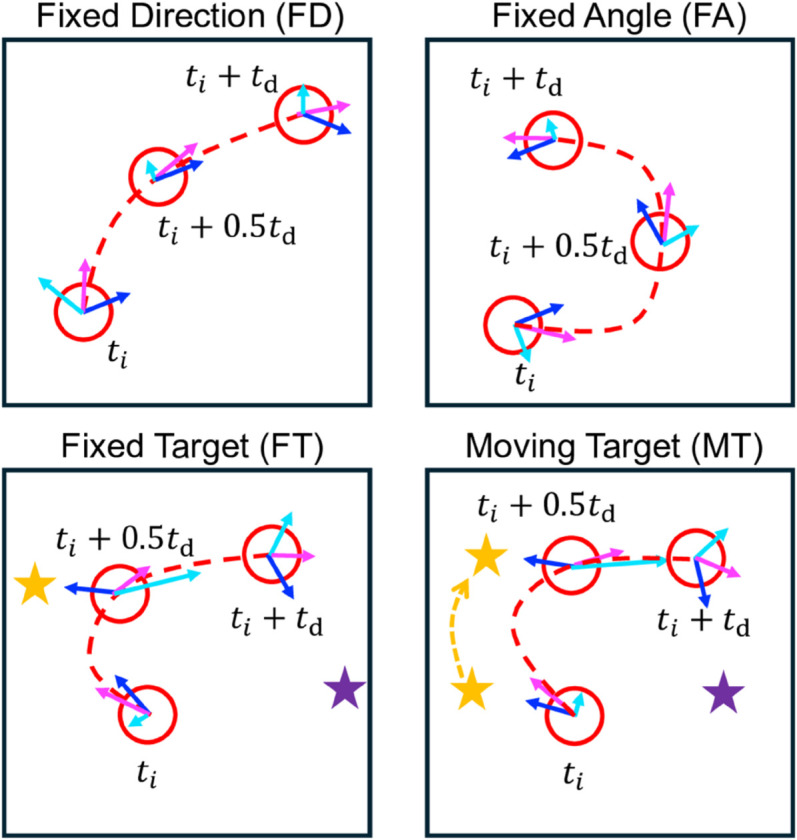
Four methods for determining the direction of ${\vec{v}}_{rel}(t)$ for the collector (blue arrow). MC velocity in the lab frame (domain) is ${\vec{v}}_{MC}=\vec{v}+{\vec{v}}_{rel}$ (magenta arrow), where $\vec{v}$ (cyan arrow) is the fluid velocity at the MC center. Stars indicate locations of fixed and moving targets for the $t_{i}<t<t_{i}+t_{d}$ (gold) and $t_{i}+t_{d}<t<t_{i}+2t_{d}$ (purple) *i* and *i* + 1 motion intervals, respectively, since $t_{i+n}=t_{i}+nt_{d}$.

The initial direction of ${\vec{v}}_{rel}$ is set by the direction of maximum capture at the start of each motion interval as described above, but the direction of ${\vec{v}}_{rel}$ during the motion interval varies according to the four different motion strategies, illustrated in Fig 4. For the fixed direction strategy (FD), ${\vec{v}}_{rel}$ points in the initial direction for the entire motion interval. For the fixed angle strategy (FA), the initial angle between ${\vec{v}}_{rel}$ and the flow $\vec{v}$ is maintained during the motion interval. The two remaining strategies, instead of maintaining fixed absolute or relative angles, continuously point the relative velocity vector at a target (stars in Fig 4) for the duration of the motion interval. For the fixed target strategy (FT), ${\vec{v}}_{rel}$ is directed at the fixed point at the end of the capture path determined in step one of the algorithm. This is akin to a ship constantly aimed at a different fixed buoy during each interval. For the moving target strategy (MT), ${\vec{v}}_{rel}$ is initially directed at the same point as in the FT approach, but here the target is advected with the flow. This is like a ship chasing a different floating object carried by the current during each motion interval. Note that all strategies become equivalent when $t_{d}\to 0$.

With the flow and MC parameters set, and one of the four motion strategies selected, the domain is initialized with 20,000 tracer particles placed uniformly on a 100×200 grid and then premixed by the flow for a duration of 20 periods (except as noted). The trajectories of all tracer particles and the MC are computed simultaneously with adaptive timesteps using the MATLAB solver ODE45 with relative and absolute tolerances of 10^−8^, for which the resulting average integration timestep is approximately 10^−4^ of the motion interval, $t_{d}$. Tracer particles that intersect the collector are removed from the flow. See S1 File in Supplementary Information for details.

## Results

### Collector trajectories

**Fig 5:**
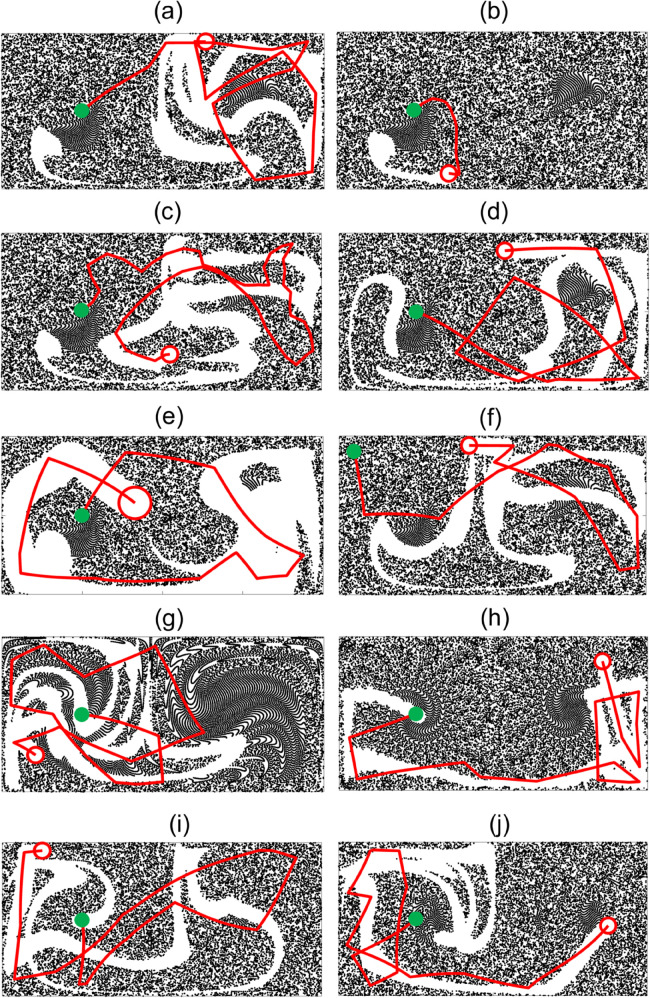
Examples of flow capture in the double-gyre flow showing MC trajectory (red) and tracer particle locations for a capture duration, $t_{total}$, equal to one period T=2π/ω=1 using the FD method. (a) A=π/2, ω=2π, ϵ=0.15, δ=0.1, $v_{rel}=5$, $t_{d}=0.1$, *L* = 0.2, start location (0.5,0.5), and a premixing time of 20. Remaining examples vary one of the parameters in (a) as noted: (b) $v_{rel}=0.5$, (c) $t_{d}=0.02$, (d) *L* = 0.5, (e) δ=0.2, (f) start location (0.1,0.9), (g) premixing time of 1, (h) A=π/10, (i) ϵ=0.01, and (j) ω=π.

Capture effectiveness potentially depends on multiple factors, including MC parameters (*δ*, $v_{rel}$), capture method parameters ($t_{d}$, *L*) and strategy, flow parameters (*A*, *ω*, *ε*), and MC initial starting location and tracer particle premixing duration. Fig 5 broadly illustrates the effects of these parameters on capture using the fixed direction (FD) method over one flow period, where the green dots indicate the MC starting location, and the red open circles indicate the MC size and location after one flow period. The collector path, which starts at (0.5, 0.5) for all but one parameter combination, is also shown in red. Although details of each trajectory vary with the parameter choices, for most parameter combinations, the MC trajectories extend into both gyres and contact the domain boundary several times. The MC trajectories and the paths cleared of particles after one flow period do not typically coincide because the underlying flow is continually moving particles after the collector has passed. Continuing the capture process for multiple flow periods (not shown) results in more particles captured, but the capture rate decreases as the number of particles in the domain decreases.

With regard to specific trajectory changes associated with parameter variation relative to the base set of values used in Fig 5(a), 5(b) shows that when $v_{rel}$ is reduced by a factor of ten, the trajectory is commensurately shorter and the MC remains in the left gyre. When the motion interval duration is reduced by a factor five to $t_{d}=0.02$, the MC changes direction more frequently as shown in (c), but still reaches both gyres. When the observation distance is increased from *L* = 0.2 to *L* = 0.5, (d) indicates few qualitative changes in the trajectory. Doubling the MC diameter *δ* creates wider paths and removes more particles (e), while changing the start location to the upper left corner (f) or decreasing the premixing time by a factor of 20 (g) causes little qualitative difference. Decreasing the flow field velocity *A* by a factor of five decreases the advection of the MC resulting in straighter paths within capture intervals (h), while making the flow less chaotic by reducing *ε* (i) and decreasing the flow oscillation frequency *ω* by a factor of two (j) again do not qualitatively affect the trajectories.

Fig 5 indicates that changing any of the eight parameters affects the trajectory of the MC and, as a result, the quantity of particles captured during the total capture time, $t_{total}$. The most influential parameters are the MC velocity relative to the flow, $v_{rel}$, and the MC diameter, *δ*. The initial direction of the collector differs from case to case except in (a-c, e) where it is the same since the distribution of particles and the observed area are the same. The other parameters affect the results, but in less obvious ways. Hence, the challenge at this point is to determine an approach to compare the impact of these various parameters on the effectiveness of the particle capture process.

To better understand the impact of the collector and flow parameters over longer capture times, this paper focuses on the influence of the collector relative velocity, $v_{rel}$, and the flow parameter *ε*, which determines how much of the flow is chaotic. The observation distance, *L*, is fixed at 0.5 and the motion duration is set to $t_{d}=L/v_{rel}$ so that the displacement of the MC relative to the fluid, $v_{rel}t_{d}$, equals the observation distance, *L*.

**Fig 6:**
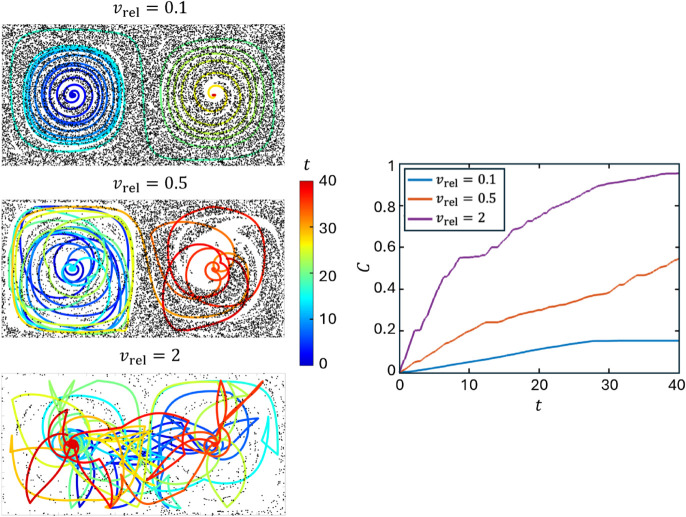
Trajectories and capture fraction, C(t), for the FA strategy with relative velocities $v_{rel}\in \{0.1,0.5,2\}$ for capture duration $t_{total}=40$ (indicated by the colorbar). A=π/2, ϵ=0.01, ω=2π, δ=0.1, *L* = 0.5, $t_{d}=L/v_{rel}$.

Consider first the effects of the MC relative velocity for $v_{rel}\in \{0.1,0.5,2\}$ and ϵ=0.01 (small chaotic region, see Fig 2) using the fixed angle (FA) strategy and shown in Fig 6, which compares the MC trajectories, colored to indicate time (0≤t≤40), the final tracer particle distributions and, in the bottom panel, the capture fraction, *C*(*t*), defined as the cumulative fraction of captured particles at time *t*, where *t* is measured in units of the flow period, *T*. For the lowest relative velocity, $v_{rel}=0.1$, the MC closely follows the underlying flow, slowly spiraling out from the center of the left gyre and then spiraling into the center of the right gyre at t≈30 where it remains for the rest of the simulation. Similarly, *C*(*t*) increases linearly until reaching ≈0.17 at t≈28 after which it remains constant. The near linear increase in *C* for *t* < 28 indicates that the MC trajectory encounters a nearly constant tracer particle density.

The situation is quite different for the highest relative velocity, $v_{rel}=2$. The MC moves throughout the domain with significantly less influence from the underlying flow compared to $v_{rel}=0.1$ and captures nearly 95% of the tracer particles. Between these extremes, for $v_{rel}=0.5$, the MC is partially influenced by the flow, evident by its spiraling trajectories in both gyres, and captures about half of the tracer particles.

The examples shown in Fig 6 confirm the strong and expected influence of $v_{rel}$ on the capture rate and demonstrate that a more informative approach is to compare the capture fraction for different $v_{rel}$ and other parameters at equal trajectory lengths relative to the fluid instead of equal capture times. This can be accomplished by scaling the total capture time by $v_{rel}$, giving the relative displacement between the MC and the fluid, and is the approach adopted in the next section.

### Capture efficiency

**Fig 7:**
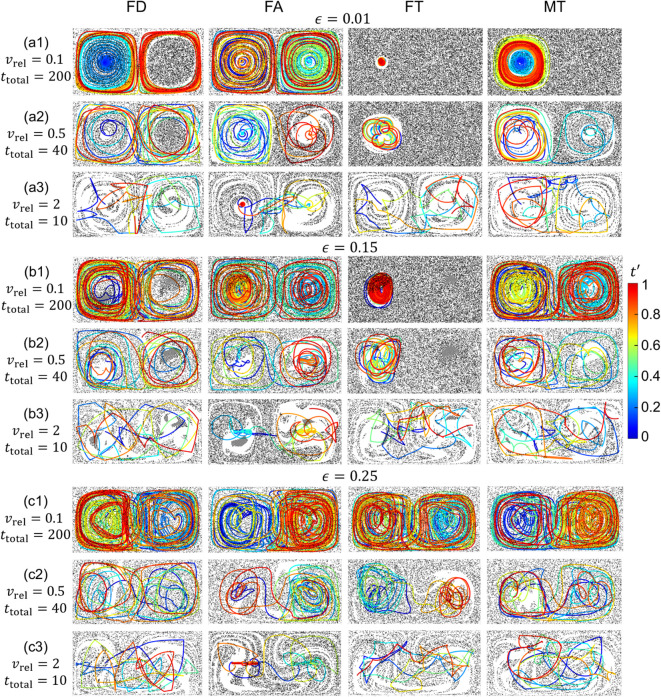
MC trajectories and final tracer particle distributions for the four capture methods (columns) and $v_{rel}\in \{0.1,0.5,2\}$ (rows) with ϵ∈{0.01,0.15,0.25}. A=π/2, ω=2π, δ=0.1, *L* = 0.5, $t_{d}=L/v_{rel}\in \{5,1,0.25\}$, $v_{rel}t_{total}=20$.

**Fig 8:**
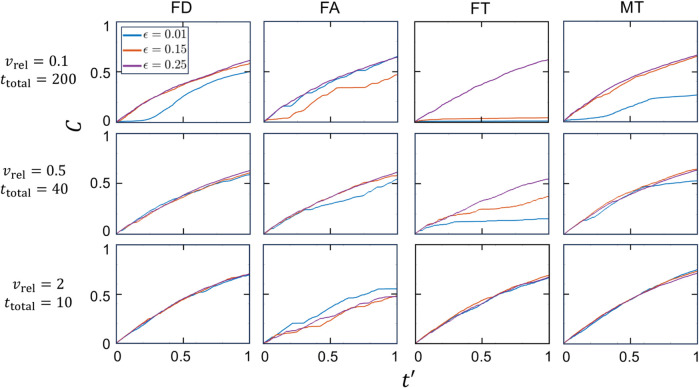
Capture fraction, C, vs. scaled time, $t^{\prime }=t/t_{total}=tv_{rel}/20,$ for the four capture methods (columns), $v_{rel}\in \{0.1,0.5,2\}$ (rows) and ϵ∈{0.01,0.15,0.25} (colors, see legend). A=π/2, ω=2π, δ=0.1, *L* = 0.5, $t_{d}=L/v_{rel}\in \{5,1,0.25\}$.

In this section, the dependence of MC trajectories and capture fraction on all four capture strategies is examined for $v_{rel}\in \{0.1,0.5,2\}$, and the fraction of the domain containing chaotic flow. To vary the degree of chaos in the flow, three values of ϵ∈{0.01,0.15,0.25} with increasing chaotic fractions are considered, as shown in the corresponding Poincaré maps in Fig 2. MC trajectories and tracer particle distributions for all 36 combinations are plotted in Fig 7. To compare the capture performance for various $v_{rel}$, the total capture time, $t_{total}$, is adjusted to keep constant the displacement of the MC relative to the moving fluid, $v_{rel}t_{total}$. Here $v_{rel}t_{total}=20$ is used for all cases. To quantify the capture process with respect to equal relative displacements of the MC to the fluid, the capture fraction, *C*, is plotted with respect to a rescaled time $t^{\prime }=t/t_{total}=tv_{rel}/20$ in Fig 8 for the same parameter combinations as in Fig 7.

First consider the twelve MC trajectories with the lowest relative velocity of $v_{rel}=0.1$ and shown Fig 7(a1, b1, c1). The most noticeable difference between these trajectories is that eight trajectories cover most of the domain while four do not. Three of the localized trajectories appear to be trapped in the left gyre in which they start (FT and MT for ϵ=0.01 and FT for ϵ=0.15), while one fully explores the left gyre but does not enter the non-chaotic region of the right gyre (FD for ϵ=0.01). Three of the four localized trajectories occur in the least chaotic flow (ϵ=0.01), while all four trajectories in the fully chaotic flow (ϵ=0.25) explore nearly the entire domain as would be expected since the MC motion differs little from the passive tracer particles at low $v_{rel}$. The corresponding capture fraction time dependence (first row of Fig 8) of seven of the eight cases that explore most of the domain are nearly identical, increasing smoothly with decreasing slope to reach C≈0.6 at $t^{\prime }=1$, with the exception of the FA method for ϵ=0.15 which exhibits a stepped increase in $C(t^{\prime })$, indicating intervals where the MC is not capturing particles. Of the four trajectories that appear trapped, $C(t^{\prime })$ is quite small for the two FT cases (ϵ∈{0.01,0.15}). The other two cases appear to have plateaus. The MT method at ϵ=0.01 exhibits an initial slow increase and a single step to a plateau, while the FD method with ϵ=0.01 appears to increase to a plateau and then increases similar to the untrapped cases for $t^{\prime }>0.2$, indicating that the MC may not be trapped.

At the highest relative velocity of $v_{rel}=2$, see Fig 7(a3, b3, c3), none of the twelve MC trajectories appear to be trapped and the corresponding capture fractions in the last row of Fig 8 increase smoothly to C≈0.7 with the exception of the FA strategy results. For the FA strategy, the trajectories are denser around *y* = 0.5, although excursions occur that reach the domain border for all three values of *ε*, and $C(t^{\prime })$ increases in a step-like fashion. Note that the tracer particles for $v_{rel}=2$ are less well-mixed than for $v_{rel}=0.1$, as evidenced by the residual cleared paths in all cases. This greater heterogeneity results from the reduced absolute time associated with scaling the simulation duration to maintain a constant path length of the MC relative to the fluid.

Finally, for the intermediate case of $v_{rel}=0.5$ two of the FT method cases remain in the left gyre, see Fig 7(a2, b2, c2). However, only for the case with ϵ=0.01 does $C(t^{\prime })$ in Fig 8 appear to hit a plateau as the trajectory stays within the non-chaotic island associated with gyre. In the case with ϵ=0.15, the MC moves between the much smaller non-chaotic island and the chaotic region [see Fig 2(b)], allowing it to capture tracer particles from the large chaotic flow regime. The other ten trajectories move between and within both gyres and the capture fraction increases relatively smoothly to $C(t^{\prime })\approx 0.6$ in all but two cases with ϵ=0.01. In the MT strategy case, $C(t^{\prime })$ increases more slowly for $t^{\prime }≳0.5$ as the MC, after a brief excursion to the right gyre, remains in the left gyre which is largely cleared of tracer particles. In the other case, which uses the FA method, the capture rate drops significantly near $t^{\prime }\approx 0.3$ but then picks up again, reaching nearly C≈0.5 by the end of the simulation as the MC finally escapes from the left gyre and enters the right gyre at $t^{\prime }\approx 0.8$.

From these results the following conclusions can be made. First, if the MC accesses the full domain it will capture more particles. Full access occurs for high values of $v_{rel}$ and when the flow is fully chaotic for all capture strategies, although the FA strategy exhibits intervals where it is temporarily localized near the gyre center under these conditions. Second, if the flow is fully chaotic, the MC should always eventually capture all tracer particles regardless of how much of the domain it covers, a result also observed for stationary collectors in previous work [25]. However, the highest capture rates and values at the end of the simulation occur in fully chaotic flows with the highest $v_{rel}=2$. Third, incomplete capture occurs for non-fully chaotic flows when the MC accesses only one gyre, as material from within the non-chaotic island of the second gyre is never captured. If the MC enters the chaotic flow region, it will ultimately capture all tracers in the chaotic region in addition to tracers in the non-chaotic island, whereas if the MC remains entirely within a non-chaotic island, the final capture fraction will be significantly less than one.

While it it clear from the examples in this section that trajectories accessing the entire domain eventually capture all of the tracer particles, it remains unclear whether the trajectories that do not explore the entire domain in non-fully-chaotic flows can eventually do so over the course of longer capture durations, $t_{total}$, or with modification of the parameters that are held constant in this section (i.e., the start location, and observation time, $t_{d}$). In the next section some of these possibilities are explored and the mechanics behind parameters that result in trapped trajectories in representative cases are explained.

### Trapped MC trajectories

**Fig 9:**
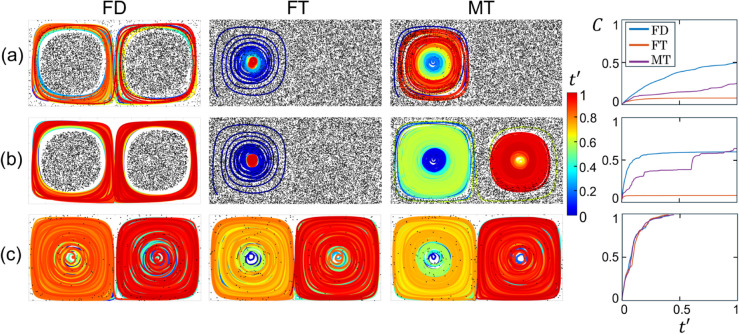
Trajectories for three capture strategies (columns 1-3) and capture fraction, C, vs. scaled capture time, $t^{\prime }$, (column 4) with parameters modified from those in Fig 7(a1) (i.e., $v_{rel}=0.1$, ϵ=0.01, $t_{total}=200$, A=π/2, ω=2π, δ=0.1, L=0.5, $t_{𝐝}=5$). (a) Different start location (0.1, 0.1). (b) Longer capture time $t_{total}=2000$ with start location as in (a). (c) Shorter observation time $t_{d}=0.1$ with start location and capture time as in (b).

To better understand the origin of trajectories that fail to explore the full domain, low $v_{rel}=0.1$, small ϵ=0.01 and the FD, FT and MT methods are considered where the MC appears to be trapped in one gyre (FT and MT) or only reaches the periphery of the other (FD), as shown in Fig 7(a1). First, the starting location of the MC is changed from the center of the left gyre (0.5, 0.5) to the lower left corner of the domain (0.1, 0.1). The resulting MC trajectories are shown in the first row of Fig 9. For the FD method, the MC is trapped in the periphery of the domain and only reaches the periphery of the non-chaotic region of both gyres, similar to the case starting at (0.5, 0.5), which spirals outward and then explores the periphery as well. For the FT method, the collector spirals into the center of the gyre and is trapped there as for the case starting at (0.5, 0.5). The MT method differs from the FD or FT method in that the MC spirals into the center of the gyre during the first several periods, spirals outward and eventually traverses a similar region to the case starting at (0.5, 0.5). Thus, in all three cases, the MC trajectories appear to converge to attractors that are subsets of the full domain, at least for the two initial conditions that are tested.

Extending the capture time by a factor of 10 to $t_{total}=2000$, corresponding to a relative trajectory length of 200 as shown in the second row of Fig 9, leads to similar trajectories for the FD and FT methods, which again appear to converge to attractors of the motion. For the MT method, in contrast, the MC first spirals into the center of the left gyre but eventually spirals back out and crosses to the other side of the domain, reaching the center of the right gyre before spiraling out again. Hence, the MC under the MT strategy has the capability to remove particles from both gyres in the long run, even though it spends long stretches in each gyre.

**Fig 10:**
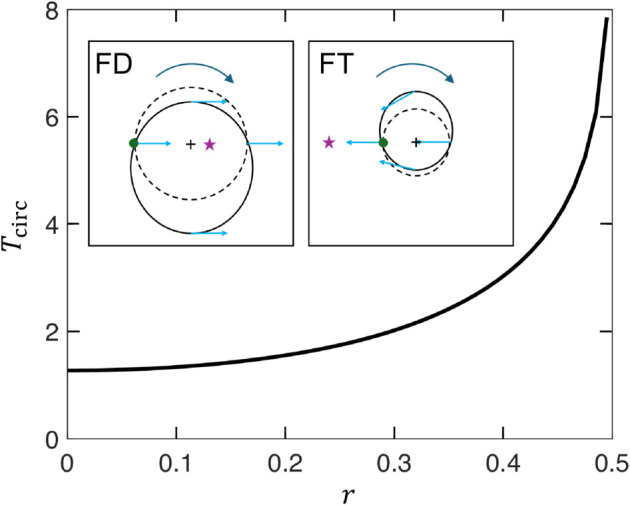
Gyre circulation time, $T_{circ}$, vs. radial distance from gyre center, r, (see text) for ϵ=0 and A=π/2. Sketches indicate trapping mechanisms for FD (left) and FT (right) methods in the left gyre. Green dots indicate MC position at start of capture interval, purple stars show direction of maximum capture (initial direction of ${\vec{v}}_{rel}$ is from green dot to purple star), dashed circles indicate purely advected trajectories ($v_{rel}=0$) about gyre center (+) and solid ellipses indicate actual trajectories which move the MC closer to the radius of the maximum capture point for $t_{i}<t⪅t_{i}+T_{circ}/2$ and further from radius of maximum capture point for $t_{i}+T_{circ}/2⪅t⪅T_{circ}.$

The circulatory motion associated with each gyre lies at the heart of the trapping phenomena. The key property for understanding the trapping mechanisms is the vortex circulation time of a tracer particle, $T_{circ}$, which is calculated using Eq 1 and expressed in units of *T*. Fig 10 plots $T_{circ}$ vs. the “radial” position *r* [measured along a line of constant *x* or constant *y* running from the gyre center at (0.5, 0.5) to the nearest domain boundary and expressed in units of the 2×1 domain size] for the simplest, but representative, case of ϵ=0 with A=π/2. The circulation time increases continuously from 4 (or, in general, 2*L*_*y*_/*A*) at the gyre center until it diverges at the edge of the domain where $\vec{v}=0$ in the corners of each gyre. When the capture interval $t_{d}$ is on the order of or greater than $T_{circ}$ in a low chaos flow and $v_{rel}$ is small, specifically $v_{rel}T_{circ}<<L_{y}$, the collector position at the end of the capture interval, $t_{d}$, can be displaced radially in the opposite direction to the initial radial component of $v_{rel}$. That is, if the direction of maximum capture lies inward toward the center of the gyre, the MC can instead move outward and vice versa depending on the relative magnitudes of $t_{d}$ and $T_{circ}$.

Consider, for example, the FD cases in Fig 9(a), 9(b) where the MC starts at (0.1, 0.1). If, at the start of any capture interval, the maximum capture direction lies inward from the initial MC position at radius *r*_0_ and toward the gyre center as sketched in the inset for FD in Fig 10, $v_{rel}$ (blue arrow) will point from the MC (green dot) towards the maximum capture location (purple star). Noting that since $v_{rel}$ is small, the MC is generally carried along with the flow (indicated by dashed circle) as it also initially moves toward the maximum capture location and a smaller radius nearer to the gyre center (+) for $t⪅T_{circ}/4$. Since the MC direction ${\vec{v}}_{rel}$ remains fixed in the FD method (${\vec{v}}_{rel}$ points left to right in the sketch), the MC then moves away from the gyre center for $T_{circ}/4⪅t⪅3T_{circ}/4$ (returning to its starting radius at $t=T_{circ}/2$ and then moving further from the gyre center for $t>T_{circ}/2$), back toward *r*_0_ for $3T_{circ}/4⪅t⪅T_{circ}$ and so on following a roughly elliptical trajectory. Whether the MC moves to a smaller or larger radius is then determined by where on the elliptical trajectory it stops, which in turn is determined by $t_{d}\mathrm{mod}T_{circ}$. If $0<(t_{d}\mathrm{mod}T_{circ})/T_{circ}<0.5$, the MC will move inward, whereas if $0.5<(t_{d}\mathrm{mod}T_{circ})<1$, the MC will move outward. For example, since $t_{d}=5$ for the data in Fig 9(a), 9(b) and using $T_{circ}$ from Fig 10, this criteria indicates that an MC with $0.3⪅r_{0}⪅0.4$ will move outward when it is trying to move inward. This result matches well with the radius of the “barrier" surrounding the uncaptured particles in the gyres shown in Fig 9(a), 9(b).

A similar effect occurs for the the FT strategy, but in this case when the fixed target is located at a larger radius, the MC ends up moving to smaller *r* because near the center $T_{circ}=4/\pi \approx 1.3$ such that $t_{d}\mathrm{mod}T_{circ}=1.1=0.85T$ as illustrated in the inset for FT in Fig 10. As a result, the MC spirals inward toward the center of the gyre, as evident for the FT strategy in Fig 9(a), 9(b).

For the MT method, where the MC explores most of the domain for $t_{total}=2000$, as shown in Fig 9, but is trapped in each gyre for t~1000, the trapping mechanism is again similar, but now involves the moving target which circulates with the gyre and is relevant only when the target is at a larger radius than the MC. In this case, the relevant time is the difference between the MC circulation time at *r*_0_ and the target circulation time, $\Delta T_{circ}$. When $t_{d}\mathrm{mod}\Delta T_{circ}<\Delta T_{circ}/2$, the MC displacement after $t_{d}$ is inward rather than outward towards the target. Note that with the MT strategy, the same effect does not occur when the target is at a smaller radius than the MC as the MC is always pointed inward toward smaller radii.

**Fig 11:**
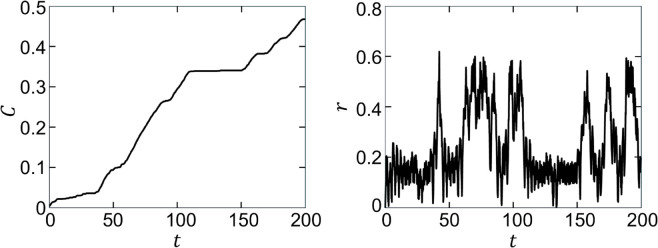
Correlation between (left) capture fraction, C(t), and (right) radial distance to closest gyre center, r(t), for $v_{rel}=0.1$, ϵ=0.15, $t_{total}=200$, A=π/2, ω=2π, δ=0.1, L=0.5, $t_{𝐝}=5$, FA method. *C* increases only when *r* is large — i.e., when the MC is not near the gyre center).

A related trapping mechanism in which *C* remains nearly constant also occurs for the FA strategy, even though the MC is only trapped intermittently near the gyre center. If the radial component of ${\vec{v}}_{rel}$ is directed inwards at the start of the capture interval, then the MC spirals into the center of the gyre as it always has a radially inward component which is maintained because the flow is mostly circular. Once near the center, r≈0, the MC only moves outward when ${\vec{v}}_{rel}$ has an outward radial component at the start of the capture interval, requiring, minimally, that the MC be directed toward a concentration of tracer particles on the same side of the gyre center as itself. Even when this condition is met, ${\vec{v}}_{rel}$ can be nearly parallel to the flow velocity, $\vec{v}$, so that the MC slowly spirals outward. An example of the correlation between the proximity of the MC to the nearest gyre center and the “steps" in *C*(*t*) (intervals when dC/dt≈0) is shown in Fig 11, where the high frequency oscillations correspond to each time the MC orbits the gyre. When the MC is near the gyre center (small *r*), *C* increases slowly or not at all. When the MC is further from the gyre center, *C* increases more quickly.

Based on the gyre trapping mechanisms described above, a possible solution for reducing or eliminating trapping is to decrease the observation time such that $t_{d}<\min(T_{circ})/2=2/\pi$. To test this, the observation time of the MC is reduced to $t_{d}=0.1<2/\pi \approx 0.64$ with the other parameters remaining the same. This allows the collector to move between different gyres and capture most of the pollutant particles for all three methods, FD, FT or MT, as shown in the third row of Fig 9. However decreasing $t_{d}$ also has the disadvantage that it requires more frequent sensing, and potentially a higher operating cost. Note that the four motion strategies are equivalent in the limit of short $t_{d}.$

## Scaling

The previous section provides insights into the impact of various parameters choices via particular examples, but provides less specific guidance for determining optimal parameters for tracer particle capture due to the large dimensionality of the full parameter space. Here a scaling approach is implemented to better understand particle capture under non-trapping conditions as explained in the previous section. To simplify the problem, the premixing time is kept constant since the pre-capture flow simply mixes the initially uniform distribution of tracer particles, which remain homogeneous in the absence of particle capture. The start location is also excluded from the varied parameters because the previous section demonstrates that its overall influence is limited to low relative velocities with short total capture times and trapped trajectories. With these parameters omitted, the combined flow (Eq 1) and MC system is characterized by two sets of parameters.

- Flow: velocity field amplitude, *A*, vortex oscillation amplitude, *ε*, vortex oscillation frequency, *ω*, and domain size, *L*_*y*_
- Collector: velocity of the MC relative to the fluid, $v_{rel}$, motion interval duration, $t_{d}$, observation distance, *L*, and MC diameter, *δ*.

**Fig 12:**
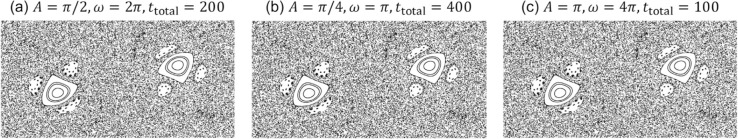
Poincaré maps with ϵ=0.1, Ω=4, and N=200 (map iterations) demonstrating flow invariance for various combinations of A and *ω* with *L*_*y*_ = 1.

First, consider the flow field. Although the position variables (*x* and *y*) are already expressed non-dimensionally on the 2×1 domain, the equations for the double-gyre flow are simplified by choosing a characteristic velocity scale, *A*, and a characteristic length scale, *L*_*y*_, with a corresponding time scale of *L*_*y*_/*A*. The resulting scaled time is $t^{*}=\frac{A}{L_{y}}t$ and the scaled velocities are $v_{x}^{*}=v_{x}/A$ and $v_{y}^{*}=v_{y}/A$. Eq 1 is then expressed as

$$v_{x}^{*}=-\sin(\pi f)\cos(\pi y),$$ (2a)

$$v_{y}^{*}=\cos(\pi f)\sin(\pi y)\frac{\partial f}{\partial x},$$ (2b)

$$f=\epsilon \sin(\Omega t^{*})x^{2}+[1-2\epsilon \sin(\Omega t^{*})]x,$$ (2c)

where $\Omega =L_{y}\omega /A$ is the ratio of the gyre oscillation frequency to the characteristic gyre circulation frequency, *A*/*L*_*y*_. With the flow equations in this form, it is clear that the flow is determined by just two parameters: *ε* and Ω. Note that although the double-gyre flow is frequently used as a canonical chaotic flow model, we are unaware that this flow invariance based on the relationship between *A* and *ω* has been previously noted. Thus, with fixed *ε* and a fixed number of map iterations, $N=\Omega t^{*}/(2\pi )$, the Poincaré map is identical for all combinations of *A*, *L*_*y*_ and *ω* that keep Ω constant. As an example, Fig 12 shows Poincaré maps with ϵ=0.1 and *N* = 200 for three different combinations of *A* and *ω* (constant *L*_*y*_) for which Ω=4. As expected, the three Poincaré maps are identical. Note that the maps are created by plotting the tracer particle positions every oscillation period, T=2π/ω, for a total time of *t* = *NT*, corresponding to scaled quantities of $T^{*}=2\pi /\Omega =2\pi^{2}A/(L_{y}\omega )$ and $t^{*}=NT^{*}$, respectively.

Similarly, the four collector parameters can also be expressed in terms of *L*_*y*_ and *A*:

$$t_{d}^{*}=\frac{t_{d}A}{L_{y}},v_{rel}^{*}=\frac{v_{rel}}{A},\delta^{*}=\frac{\delta }{L_{y}},L^{*}=\frac{L}{L_{y}}.$$ (3)

In this form, $t_{d}^{*}$ represents the ratio between the duration of the capture strategy motion interval, $t_{d}$, and a characteristic flow circulation period *L*_*y*_/*A* as mentioned in the context of Fig 10. For $t_{d}^{*}\ll 1$, the tracer particle distribution changes minimally during the capture interval, whereas for $t_{d}^{*}\gg 1$, tracers are redistributed by the shearing action of the gyres and, depending on *ε*, the degree of chaos in the flow. Similarly, $v_{rel}^{*}$ represents the relative velocity of the collector to the flow. For $v_{rel}^{*}\gg 1$, the collector moves through an effectively stationary distribution of particles. For $v_{rel}^{*}\ll 1$, the MC is largely driven by the flow, which can lead to trapped MC trajectories when $t_{d}$ is on the order of or larger than $T_{circ}$ as discussed above.

**Fig 13:**
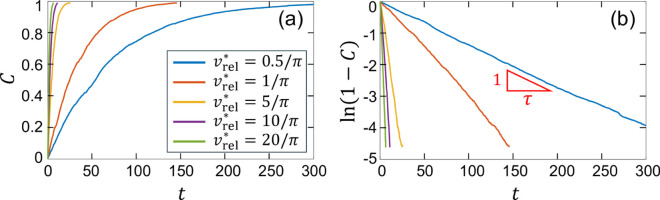
(a) Capture fraction, C, and (b) ln(1−C) vs. time, t, under the FD method for relative MC velocities $\pi v_{rel}^{*}=1/2,1,5,10,20$. The slopes of the curves in (b) correspond to the inverse of the capture time scale, *τ*, as defined in Eq 4. Flow parameters: A=π/2, ω=2π, ϵ=0.15, *L*_*y*_ = 1, Ω=4. Collector parameters: $L^{*}=0.5$, $t_{d}^{*}=0.5$, $\delta^{*}=0.1$.

To quantify the particle capture process and its parameter dependence, the capture fraction, *C*, versus time, *t*, is measured as previously described for specific cases in the contexts of Figs 6 and 8. Typical examples of *C*(*t*) under the FD method for times sufficiently long to capture nearly all of the tracer particles are shown in Fig 13 for cases where the collector is untrapped. For a fixed observation distance $L^{*}=0.5$, motion interval $t_{d}^{*}=0.5$, and collector diameter $\delta^{*}=0.1$, the MC velocity is varied from $v_{rel}^{*}=1/(2\pi )$ to $v_{rel}^{*}=20/\pi$. For the first few flow periods (noting that *T* = 1), *C*(*t*) is approximately linear but then increases more slowly as time increases. This behavior occurs because at short times the particle density is nearly constant regardless of the MC’s path, and C∝t. At later times, the capture rate, *dC*/*dt*, decreases since fewer particles remain in the domain, which decreases the average particle density. It is evident that for the range of relative velocities considered in the figure, nearly all the tracer particles are eventually captured (C→1), and the time for this to occur decreases with increasing $v_{rel}$.

Fig 13(a) indicates that *C*(*t*) depends exponentially on time, i.e.,

$$C(t)=C_{max}(1-e^{-t/\tau }),$$ (4)

where *τ* is the characteristic time scale for capture. (Note that in our paper on SC capture [25], *τ* was incorrectly indicated as 1/τ in Fig 11(b,c) and Fig 13 as discussed in the associated erratum.) Eq 4 has the same functional dependence on time as for a stationary collector [25], however, unlike the stationary capture problem where $C_{max}<1$ for all flows that are not fully chaotic, $C_{max}=1$ for an untrapped MC in all cases since all particles are eventually captured. This allows Eq 4 to be rearranged as ln[1−C(t)]=−t/τ. Plotting *C*(*t*) in this form in Fig 13(b) indicates that Eq 4 accurately models the capture process and allows *τ* to be determined as the inverse of the slope. Smaller $v_{rel}^{*}$ values correspond to lower slopes and longer capture times, and vice versa, as expected.

Eq 4 describes a capture process for which the capture rate is proportional to the amount of material remaining in the domain, 1–*C*(*t*), [i.e., $\frac{dC}{dt}=(1-C)/\tau$] and for which the tracer particles remain effectively well-mixed along the path of the MC (noting that the tracer concentration is clearly depleted immediately behind the MC as shown in Fig 7, a point considered further below. We hypothesize that the timescale *τ* is proportional to the domain area, $2L_{y}^{2}$, divided by the areal fluid flux into the MC, Φ. When $v_{rel}^{*}$ is large, Φ is determined by the relative velocity and diameter of the MC such that the “motion”-flux is $\Phi_{m}=v_{rel}\delta$. However, when $v_{rel}^{*}$ is small, the capture flux is dominated by the velocity gradient across the MC, such that the “gradient”-flux is $\Phi_{g}=\dot{\gamma }\delta^{2}$, where $\dot{\gamma }\propto A/L_{y}$ is representative of the velocity gradient across the MC. Thus, the corresponding timescales for the motion-flux and the gradient-flux are $\tau_{m}=\frac{2L_{y}^{2}}{v_{rel}\delta }$ and $\tau_{g}=\frac{2L_{y}^{3}}{A\delta^{2}}$, respectively. Scaling both terms by the characteristic time *L*_*y*_/*A* gives $\tau_{m}^{*}=2/(v_{rel}^{*}\delta^{*})$ and $\tau_{g}^{*}=2/\delta^{*2}.$ Since the motion and gradient capture processes act simultaneously, $C(t)=1-e^{-t/\tau_{m}}e^{-t/\tau_{g}}$. Hence, the overall capture timescale is $\tau =\frac{\tau_{m}\tau_{g}}{\tau_{m}+\tau_{g}}$, and the scaled capture time is

$$\tau^{*}=\frac{\tau_{m}^{*}\tau_{g}^{*}}{\tau_{m}^{*}+\tau_{g}^{*}}=\frac{2}{\delta^{*}\left(\delta^{*}+v_{rel}^{*}\right)}=\frac{2L_{y}}{\delta \left(\frac{\delta }{L_{y}}+\frac{v_{rel}}{A}\right)}.$$ (5)

**Fig 14:**
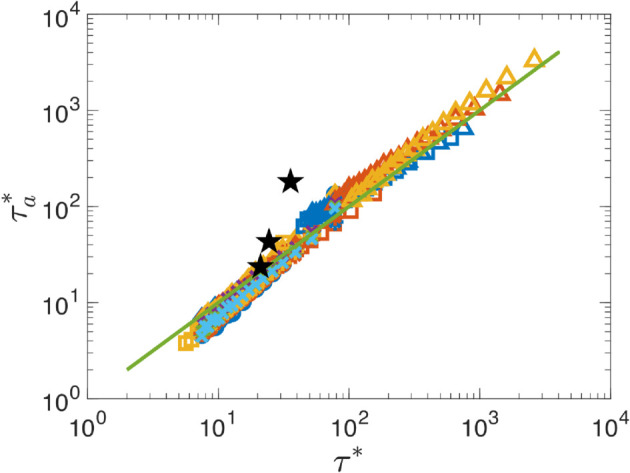
Measured scaled capture time, $\tau_{𝐚}^{*}$, vs. predicted characteristic time scale, $\tau^{*}$, as defined in Eq 5, for the 342 flow and MC parameters specified in <s002>S1 Table, and for three stationary collector locations from [25] (see text). The reference line indicates $\tau_{a}^{*}=\tau^{*}$.

To determine if the hypothesized $\tau^{*}$ in Eq 5 provides the proper scaling for particle capture in the absence of trapping, *C*(*t*) is calculated for a large range of flow and MC parameters, as well as different capture methods, as specified in S1 Table for 342 distinct simulations. For each simulation, the measured (“ **a**ctual”) value of the scaled timescale, $\tau_{a}^{*}=\tau_{a}A/L_{y}$, is determined from the capture fraction data as shown in Fig 13(b), and is plotted in Fig 14 versus the scaled characteristic capture timescale, $\tau^{*}$, as defined in Eq 5. The results, shown on a log-log scale, indicate that $\tau_{a}^{*}$ is close to linear in $\tau^{*}$ over nearly four orders of magnitude for all of the parameter combinations tested. This confirms that in the absence of trapping, the capture process is well-characterized by the kinematic and gradient timescales and that the scaling using *A* and *L*_*y*_ resulting in a time scale *L*_*y*_/*A* is appropriate. Also note that for the parameter combinations tested, the ratio of $\tau_{m}$ to $\tau_{g}$ ranges from $\approx 10^{-3}$ to 10^1^, indicating, respectively, that the kinematic and gradient dominated capture regimes are both well represented in the figure.

As mentioned in the Introduction as motivation for this study, a mobile collector should be capable of removing all tracer particles from the domain and do so more quickly than a stationary collector of the same diameter located at (*x*,*y*) when $v_{rel}>v(x,y)$. To compare the performance of MCs and SCs, Fig 14 additionally includes three data points for an SC with δ=0.1 located at (0.6,0.5), (0.8,0.5), and (1,0.5) with $|{\vec{v}}_{rel}|=|\;-\;\vec{v}(x,y)|=|v(x,y){|}_{avg}=0.7239,1.1289,1.3377$, respectively, in a fully chaotic flow (ϵ=0.25) where it is able to eventually capture all the tracer particles ($C_{max}=1)$. As for the MC, $\tau^{*}$ is calculated using Eq 5 and $\tau_{a}^{*}$ is measured from the *C*(*t*) data for the stationary collector; the results are plotted in Fig 14 using black stars. At the smallest value of $\tau^{*}=21$ for the SC, the measured $\tau_{a}^{*}$ is slightly larger than the MC values at similar $\tau^{*}$, while at the largest value of $\tau^{*}=36$, $\tau_{a}^{*}$ is approximately an order of magnitude larger than the corresponding MC values. These results demonstrate that, indeed, the MC removes tracer particles more rapidly than the SC for the same collector parameters (*δ* and $v_{rel}$), which is unsurprising given that the MC actively seeks out regions of high concentration whereas the SC must wait for those regions to be brought to it by the flow.

It is helpful to consider the practical application of the scaling developed in this section via an example at a geophysical scale. Consider using the motion-flux timescale for the double-gyre flow, $\tau_{m}=\frac{2L_{y}^{2}}{v_{rel}\delta }$, for an MC moving with $v_{rel}=1$ m/s, having a characteristic collector size of δ=100 m and operating in the Gulf of Mexico ($L_{y}^{2}\sim 2\times 10^{6}$ km^2^). These MC parameters and the size of the Gulf of Mexico domain yields a time scale of about 600 years. This implies that it would take about three years for a fleet of 1000 MCs to remove 99% of a dispersed surface pollutant from the Gulf, ignoring many other factors such as emptying, refueling and servicing the MCs, three-dimensional mixing, and capture efficiency. While many assumptions and simplifications are involved with the analysis in this paper, the scaling developed in this section provides a meaningful approach to estimate the effort necessary for clean-up operations in real geophysical flows.

Lastly, as mentioned above, the observed dependence of the capture rate, *dC*/*dt*, on 1–*C*(*t*) is indicative of a relatively well-mixed domain. In chaotic regions of the flow, our earlier work indicated that mixing occurs in times on the order of a few circulation periods [25], and this is confirmed by the degree of mixing evident in the fully chaotic flows of Fig 7(c). When the flow is not fully chaotic, the radially varying circulation time in the KAM islands (see Fig 10) allows angular mixing to occur within the islands as evident in Fig 7(a).

## Conclusions

Capturing pollutants from dynamic, chaotic environmental flows, such as exist on the ocean, using a stationary or mobile collector is far from simple. Extending the work of Wang, *et al*. [25], which focuses on a stationary collector (SC) in a model chaotic double-gyre flow, this study considers four motion strategies (FD, FA, FT, and MT), emphasizing similarities and differences between various approaches for a mobile collector (MC) motion and characterizing their effectiveness for a wide range of flow and collector parameters. The complete system involves eight parameters that influence the capture results, of which both capture parameters and flow parameters are significant. When the flow is fully chaotic (ϵ=0.25), the MC relative velocity and motion strategy make little difference, as the tracer particles can move anywhere and all particles are eventually captured. Differences between capture strategies are more evident when the flow is less chaotic and the MC has a small velocity relative to the underlying flow. In these cases, trapped MC trajectories can occur, but choosing an observation time shorter than the shortest gyre circulation period eliminates the trapping.

When trapping does not occur, the scaling of the capture fraction is explained by two timescales, the motion timescale, $\tau_{m}$, when the MC velocity is dominant, and the gradient timescale, $\tau_{g}$, when the flow velocity is dominant. The combination of these two timescales, $\frac{\tau_{m}\tau_{g}}{\tau_{m}+\tau_{g}}$, accurately reflects the scaling of the overall characteristic capture time, *τ*, for a broad range of flow and collector parameters.

This research provides insight for determining strategies for the motion of a mobile collector in chaotic flows with vortices, but the complexity of the problem involving multiple parameters deserves further study. For example, it would be worthwhile to consider using multiple MCs and varying their degree of coordination. Another idea is to consider trajectory planning when global flow and tracer particle information is known rather than just using local concentration information, or to use both global and local information together to monitor and predict the movement of tracer particles. The biggest challenge is to move beyond the simple model flows examined here and consider actual geophysical velocity fields for broader applications [9,41,42].

## Supporting information

S1 File MATLAB simulation code. (PDF)

S1 TableFlow and MC parameter combinations.Combinations of flow (columns 1-4) and MC (columns 5-7) parameters with corresponding markers for $\tau_{a}^{*}$ and $\tau^{*}$ data shown in Fig 14 where, for each parameter set (row), $0.02\leq \delta^{*}\leq 0.2$ in increments of 0.01 with *L*_*y*_ = 1 and $L^{*}=0.5$.(PDF)
