# Supplementary material for: Mobile-collector capture of particles in a chaotic flow
Source: PLoS One. 2025 Aug 7;20(8):e0329766. doi: 10.1371/journal.pone.0329766 (PMC12331103; doi:10.1371/journal.pone.0329766)
Supplement: S1 File — (PDF) [file pone.0329766.s001.pdf]

```
%% Sample Matlab code
%%%adaptive ode timestep
%%%separate ta (ode timestep) & td (observe & change direction
  timestep)
%%%move with relative velocity to water
%%%consider when hit the wall
%%%weight the same observe direction and choose the best
%%%no particle around, use previous direction
%%%FD method
close all
clear all
clc
format long
A = 0.5;
omega = 2*pi;
epsilon = 0.01;
period = 2000;
period0 = 10;
NN = period/period0;
unitR = 0.05;%unit radius
```

```
unitV_rel = 0.1;%relative velocity to water
obs_dis = 0.5;%distance L for observation (fixed)
%td = 0.1;
td = obs_dis/unitV_rel;%timestep to change direction
dx = 0.01; % interval of pollutant
[xp,yp] = meshgrid(dx/2:dx:2-dx/2,dx/2:dx:1-dx/2);%location of
   pollutant
xPos0 = reshape(xp,1,[]);%location of pollutant
yPos0 = reshape(yp,1,[]);
np0 = length(xPos0); %total number of pollutant
sigma = pi/50:pi/50:2*pi; %100 directions
nd = length(sigma); %total number of direction
start_time = 20;
m0 = 0.1;%location of start point!!!
n0 = 0.1;
unitXPOS = m0;%store unit position at each timestep
unitYPOS = n0;
T_cir = 2/(pi*A);
OPT=odeset('RelTol',1e-8,'AbsTol',1e-8);
flag = 0;
tic
%%%%start time is not zero
if start_time>0
    p0_all = [xPos0; yPos0];%2*np matrix: initial conditions
```

```
    tic
    [tt0,traj0] = ode45(@(t,p) doublegyresys(A,omega,epsilon,t,p,
       np0),[0 start_time],p0_all,OPT);
    toc
    traj0 = reshape(traj0,[],np0);
    nt0 = length(tt0);
    xPos_start = traj0(nt0,:);
    yPos_start = traj0(end,:);
else
    xPos_start = xPos0;
    yPos_start = yPos0;
end
clear p0_all tt0 traj0
%%%capture time t = 0
cap_idx0 = ifcapture(xPos_start,yPos_start,m0,n0,unitR); %indices
    of captured particles
cap_num = length(cap_idx0); %number of captured particles
xPos_start(cap_idx0) = [];
yPos_start(cap_idx0) = []; %remove the inside particle
np_new = length(xPos_start); %reduced number of pollutant
%np_new = np0;
cost_num = 0;
cap_ts = 0;
cap_result = cap_num;
```

```
cost_result = cost_num;
%%%%%plot t = 0
h = figure;
plot(xPos_start,yPos_start,'.','MarkerSize',10,'MarkerEdgeColor',
   'black');
hold on;
xCircle = [m0 + unitR*cos(sigma) m0 + unitR*cos(sigma(1))];
yCircle = [n0 + unitR*sin(sigma) n0 + unitR*sin(sigma(1))];
plot(xCircle,yCircle,'LineWidth',3,'Color','red');
Title = title(['$$t = 0T$$']);
axis equal;
axis([0,2,0,1]);
xticks([0 0.5 1 1.5 2]);
yticks([0 0.5 1]);
set([Title],'FontName', 'AvantGarde','Interpreter','latex');
set([Title],'FontSize', 15);
box on
saveas(h,sprintf('MOVIE_TEST_0.bmp'));
pause(0.5)
hold off
close
%%%%%
td_time = 0:td:period;
count_td = 1;
```

```
%%%start observe & capture
for i = 1:NN
    period_temp = i*period0;
    %calculate trajectory
    pn_all = [xPos_start; yPos_start];%2*np matrix: initial
       conditions
    tic
    [tt_n,traj_n] = ode45(@(t,p) doublegyresys(A,omega,epsilon,t,
       p,np_new),[period_temp-period0 period_temp],pn_all,OPT);
    toc
    traj_n = reshape(traj_n,[],np_new);
    nt_n = length(tt_n);
    Xx_traj = traj_n(1:nt_n,:); %column:different particles row:
       different time
    Yy_traj = traj_n(nt_n+1:end,:);
    count_tt = 1;

    dir_idx = find((td_time>=period_temp-period0) & (td_time<
       period_temp));
    dir_num = length(dir_idx);

    for k = 1:dir_num %loop for every direction change timestep
        td_temp = td_time(count_td)
        x_obs = m0 + obs_dis*cos(sigma); % observed distance
```

```
y_obs = n0 + obs_dis*sin(sigma);
obs_ind = cell(1,nd);
obs_num = zeros(1,nd);

for j = 1:nd %for each direction
    obs_ind{j} = ifobserve(Xx_traj(count_tt,:),Yy_traj(
       count_tt,:),m0,n0,x_obs(j),y_obs(j),sigma(j),unitR
       ,obs_dis); %indices of observed particle
    obs_num(j) = length(obs_ind{j}); %number of observed
       particles
end
[obs_num_max,obs_dir_ind] = max(obs_num);%value & indice
   of observed most

if obs_num_max > 0
    notation = ['obs_num = ',num2str(obs_num_max)];
    disp(notation)
    num_max = length(find(obs_num == obs_num_max)) %how
       many directions can observe most
    if num_max > 1
        max_idx = find(obs_num == obs_num_max);
        weight_dis = zeros(1,length(max_idx));
        for wi = 1:length(max_idx)
            Xx_obs_loc = Xx_traj(count_tt,obs_ind{max_idx
```

```
                (wi)});
            Yy_obs_loc = Yy_traj(count_tt,obs_ind{max_idx
                (wi)});
            weight_dis(wi) = weightdis(Xx_obs_loc,
                Yy_obs_loc,m0,n0);
        end
        [weight_dis_max,weight_dis_ind] = max(weight_dis)
           ;
        dir_idx_final = max_idx(weight_dis_ind);  %final
           moving direction index
    else
        dir_idx_final = obs_dir_ind;
    end
    %no particle around, use previous direction
end
final_sigma = sigma(dir_idx_final);
step_idx = find(tt_n>td_temp & tt_n<td_temp+td);
step_num = length(step_idx)
notation = ['sigma1 = ',num2str(final_sigma)];
    disp(notation)
for s = 1:step_num %loop for every adaptive timestep

    if (count_tt == 1) && (i == 1)
        v_water_t = 0;
```

```
elseif (count_tt == 1) && (i > 1)
    v_water_t = tt_n_end;
else
    v_water_t = tt_n(count_tt);
end
v_water = velocitysys(A,omega,epsilon,v_water_t,[m0
   n0]);
v_rel_x = unitV_rel*cos(final_sigma);
v_rel_y = unitV_rel*sin(final_sigma);
v_abs_x = v_rel_x + v_water(1);
v_abs_y = v_rel_y + v_water(2);
m0_temp = m0 + v_abs_x * (tt_n(count_tt+1)-v_water_t)
   ; %all direction unit center x position
n0_temp = n0 + v_abs_y * (tt_n(count_tt+1)-v_water_t)
   ;%all direction unit center y position
if (m0_temp<=2-unitR && m0_temp>=unitR) && (n0_temp
   <=1-unitR && n0_temp>=unitR) %not hit the wall
    m0 = m0_temp;
    n0 = n0_temp;
else %hit wall reset the direction
    %%%%repeat observe and choose direction
    x_obs = m0 + obs_dis*cos(sigma); % observed
       distance
    y_obs = n0 + obs_dis*sin(sigma);
```

```
obs_ind = cell(1,nd);
obs_num = zeros(1,nd);
for j = 1:nd %for each direction
    obs_ind{j} = ifobserve(Xx_traj(count_tt,:),
       Yy_traj(count_tt,:),m0,n0,x_obs(j),y_obs(j
       ),sigma(j),unitR,obs_dis); %indices of
       observed particle
    obs_num(j) = length(obs_ind{j}); %number of
       observed particles
end
[obs_num_max,obs_dir_ind] = max(obs_num);%value &
    indice of observed most
final_sigma = sigma(obs_dir_ind(1));
v_rel_x = unitV_rel*cos(final_sigma);
v_rel_y = unitV_rel*sin(final_sigma);
v_abs_x = v_rel_x + v_water(1);
v_abs_y = v_rel_y + v_water(2);
m0_temp = m0 + v_abs_x * (tt_n(count_tt+1)-
   v_water_t); %all direction unit center x
   position
n0_temp = n0 + v_abs_y * (tt_n(count_tt+1)-
   v_water_t);%all direction unit center y
   position
m0 = m0_temp;
```

```
    n0 = n0_temp;
end
notation = ['sigma2 = ',num2str(final_sigma)];
disp(notation)
unitXPOS = [unitXPOS m0];
unitYPOS = [unitYPOS n0];

cost_temp = unitV_rel * (tt_n(count_tt+1)-v_water_t);
cost_num = cost_num + cost_temp;
cost_result = [cost_result cost_num];

cap_idx = ifcapture(Xx_traj(count_tt+1,:),Yy_traj(
   count_tt+1,:),m0,n0,unitR);
cap_num_temp = length(cap_idx);
cap_num = cap_num + cap_num_temp
Xx_traj(:,cap_idx) = [];
Yy_traj(:,cap_idx) = [];
cap_ts = [cap_ts tt_n(count_tt+1)];
cap_result = [cap_result cap_num];

count_tt = count_tt+1

 if tt_n(count_tt)>1999.99
%%%%%%
```

```
h = figure;
plot(Xx_traj(count_tt,:),Yy_traj(count_tt,:),'.','
   MarkerSize',10,'MarkerEdgeColor','black');
hold on;
xCircle = [m0 + unitR*cos(sigma) m0 + unitR*cos(sigma
   (1))];  %%% plot unit
yCircle = [n0 + unitR*sin(sigma) n0 + unitR*sin(sigma
   (1))];
plot(xCircle,yCircle,'LineWidth',3,'Color','red');
plot(unitXPOS,unitYPOS,'LineWidth',3,'Color','m')  %
   %% plot path
%p = patchline(unitXPOS,unitYPOS,'lineWidth',3,'
   edgecolor','m','edgealpha',0.4);
xCircle2 = [m0 + obs_dis*cos(final_sigma) + unitR*cos
   (sigma) m0 + obs_dis*cos(final_sigma)+ unitR*cos(
   sigma(1))];
yCircle2 = [n0 + obs_dis*sin(final_sigma)+ unitR*sin(
   sigma) n0 + obs_dis*sin(final_sigma) + unitR*sin(
   sigma(1))];
xCircle3 = [m0 + (unitR+obs_dis)*cos(sigma) m0 + (
   unitR+obs_dis)*cos(sigma(1))];
yCircle3 = [n0 + (unitR+obs_dis)*sin(sigma) n0 + (
   unitR+obs_dis)*sin(sigma(1))];
p1 = [m0+unitR*cos(final_sigma+pi/2) n0+unitR*sin(
```

```
            final_sigma+pi/2)];
        p2 = [m0+obs_dis*cos(final_sigma)+unitR*cos(
            final_sigma+pi/2) n0+obs_dis*sin(final_sigma)+
            unitR*sin(final_sigma+pi/2)];
        p3 = [m0+unitR*cos(final_sigma-pi/2) n0+unitR*sin(
            final_sigma-pi/2)];
        p4 = [m0+obs_dis*cos(final_sigma)+unitR*cos(
            final_sigma-pi/2) n0+obs_dis*sin(final_sigma)+
            unitR*sin(final_sigma-pi/2)];
%               if s == 1  %%% plot interval circle
%                   plot(xCircle2,yCircle2,'LineWidth',2,'Color','g
   ');
%                   plot([ p1(1) p2(1)], [p1(2) p2(2)],'LineWidth
   ',2,'Color','g');
%                   plot([ p3(1) p4(1)], [p3(2) p4(2)],'LineWidth
   ',2,'Color','g');
%                   plot(xCircle3,yCircle3,'LineWidth',2,'Color','g
   ');
% %               else
% %                   plot(xCircle2,yCircle2,'LineWidth',2,'Color
   ','magenta');
% %                   plot([ p1(1) p2(1)], [p1(2) p2(2)],'LineWidth
   ',2,'Color','magenta');
% %                   plot([ p3(1) p4(1)], [p3(2) p4(2)],'LineWidth
```

```
    ',2,'Color','magenta');
% %                   plot(xCircle3,yCircle3,'LineWidth',2,'Color
    ','magenta');
%              end

            norm_v = unitV_rel/5;
            norm_water = norm(v_water)/5;
            norm_abs = norm([v_abs_x v_abs_y])/5;
            move_abs = sqrt((m0-unitXPOS(end-1))^2+(n0-unitYPOS(
               end-1))^2);
            %%find the angle between water and x axis
            vec_x =  [1,0];
            ang2 = atan2(vec_x(1)*v_water(2)-v_water(1)*vec_x(2),
               vec_x(1)*v_water(1)+vec_x(2)*v_water(2));
            ang_x_w = mod(ang2,2*pi);
            v_vec1 = [m0 m0+norm_v*cos(final_sigma)];
            v_vec2 = [n0 n0+norm_v*sin(final_sigma)];
            w_vec1 = [m0 m0+norm_water*cos(ang_x_w)];
            w_vec2 = [n0 n0+norm_water*sin(ang_x_w)];
            abs_vec1 = [m0 m0+norm_abs*((m0-unitXPOS(end-1))/
               move_abs)];
            abs_vec2 = [n0 n0+norm_abs*((n0-unitYPOS(end-1))/
               move_abs)];
```

```
            %drawArrow(v_vec1,v_vec2,{'Color','b','LineWidth',5})
               ;
            %drawArrow(w_vec1,w_vec2,{'Color','c','LineWidth',5})
               ;
            %drawArrow(abs_vec1,abs_vec2,{'Color','m','LineWidth
               ',5});

            Title = title(['$$t = $$',num2str(tt_n(count_tt)),'$$
               T$$']);
            axis equal;
            axis([0,2,0,1]);
            xticks([0 0.5 1 1.5 2]);
            yticks([0 0.5 1]);
            set([Title],'FontName', 'AvantGarde','Interpreter','
               latex');
            set([Title],'FontSize', 15);
            box on
            saveas(h,sprintf('MOVIE_TEST_%d.bmp',count_tt));
            pause(2)
            hold off
            close
% %
            end
```

```
% %               %%%%%


            if cap_num/np0 > 0.99
                %if td_temp>2
                flag = 1;
                break
            end
        end

        if (flag == 1)
            break
        end
        count_td = count_td+1;
    end

    if (flag == 1)
        break
    end

    xPos_start = Xx_traj(end,:);
    yPos_start = Yy_traj(end,:);
    np_new = length(xPos_start)
```

```
    tt_n_end = tt_n(end-1);
end
toc
x_p = Xx_traj(count_tt,:);
y_p = Yy_traj(count_tt,:);
%save('FD(0.1,0.1)t2000_ep0.01v0.1td0.1.mat','unitXPOS','unitYPOS
   ','x_p','y_p','xCircle','yCircle','cap_ts','cost_result','
   cap_result')
```
