# Supplementary material for: Mobile-collector capture of particles in a chaotic flow
Source: PLoS One. 2025 Aug 7;20(8):e0329766. doi: 10.1371/journal.pone.0329766 (PMC12331103; doi:10.1371/journal.pone.0329766)
Supplement: S1 Table — Combinations of flow (columns 1-4) and MC (columns 5-7) parameters with corresponding markers for τa* and τ* data shown in Fig 14 where, for each parameter set (row), 0.02≤δ*≤0.2 in increments of 0.01 with Ly = 1 and L*=0.5. (PDF) [file pone.0329766.s002.pdf]

A/π	ω	ϵ	Ω	t_d^*/π	πv_rel^*	Method	Marker
1/2	2π	0.01	4	1/20	4	FD	○
1/2	2π	0.15	4	1/20	4	FD	○
1/2	2π	0.25	4	1/20	4	FD	○
1/2	2π	0.25	4	1/4	4	FD	◇
1/2	2π	0.25	4	1	4	FD	◇
1/2	2π	0.25	4	2	4	FD	◇
1/2	2π	0.25	4	1/20	1/2	FD	□
1/2	2π	0.25	4	1/20	2	FD	□
1/2	2π	0.25	4	1/20	10	FD	□
1/20	2π	0.25	40	1/20	4	FD	✱
1/5	2π	0.25	10	1/20	4	FD	✱
1	2π	0.25	2	1/20	4	FD	✱
1	4π	0.25	4	1/20	2/5	FD	△
5/4	5π	0.25	4	1/20	1/5	FD	△
3/2	6π	0.25	4	1/20	1/10	FD	△
1/2	2π	0.25	4	1/20	4	FA	×
1/2	2π	0.25	4	1/20	4	FT	×
1/2	2π	0.25	4	1/20	4	MT	×

Table S1 Combinations of flow (columns 1-4) and MC (columns 5-7) parameters with corresponding markers for τ_a^* and τ^* data shown in Fig 14 where, for each parameter set (row), $0.02 \leq \delta^* \leq 0.2$ in increments of 0.01 with $L_y = 1$ and $L^* = 0.5$.
